# Upcycling of Non-Standard Cakes by Manufacturing Ring-Shaped Cookies

**DOI:** 10.3390/foods13244041

**Published:** 2024-12-14

**Authors:** Nicolle Christina Silvester Vieira Campanini, Cláudia Moreira Santa Catharina Weis, Elizabeth Harumi Nabeshima, Luciana Camargo Temoczko, Larissa Canhadas Bertan, Vania Zanella Pinto, Leda Battestin Quast

**Affiliations:** 1 Graduate Program in Food Science and Technology (PPGCTAL), Federal University of the Fronteira Sul (UFFS), Laranjeiras do Sul 85319-899, Brazil; nicolle.csv@gmail.com (N.C.S.V.C.); engalimclaudiaweis@gmail.com (C.M.S.C.W.); larissa.bertan@uffs.edu.br (L.C.B.); 2 Center of Chocolate and Cereals (Cereal Chocotec), Institute of Food Technology (Ital), Campinas 13070-178, Brazil; nabeshima@ital.sp.gov.br; 3 Food Engineering, University of Centro Oeste (Unicentro), Guarapuava 85040-167, Brazil; luciana.lct@gmail.com

**Keywords:** by-product, bakery, mini cake, sustainability

## Abstract

A creative approach to reducing food waste by reusing industrial cake waste can result in the development of a worldwide favorite snack. This study aimed to evaluate the potential of industrial cake wastes—basic mini cake (BMC), filled mini cake (FMC), and traditional mini cake (TMC)—as sustainable alternatives to wheat flour in producing ring-shaped cookies. After initial screening, FMC was upcycled at 10%, 15%, 20%, 30%, and 50% of wheat flour replacement for ring-shaped cookie processing. Only the formulations containing 10, 15, and 20% FMC waste showed good moldability and similar rheological parameters between each other (*p* < 0.05). After baking, the cookies made with FMC waste showed greater volume expansion (*p* < 0.05) compared to the standard formulation. All cookies had water activity below 0.6 and remained stable during the 150 days of storage. Furthermore, after 150 days of storage, the hardness of the standard (T1) formulation was 40.02 N, while formulations with FMC waste (10%—T2, 15%—T3, and 20%—T4) resulted in softer cookies, with hardness values of 26.9 N, 27.9 N, and 27.61 N, respectively. The ring-shaped cookies containing 15% FMC waste showed the best technological performance, considering manufacture, physicochemical, and hardness traits, with no nutritional differences compared to the control.

## 1. Introduction

Cookies represent a category of cereal-based products widely consumed worldwide [1], and Brazil is a major exporter and consumer of this product [2]. The quality of cookies is directly related to the raw materials used in the formulations, and the ingredients can have different functions depending on their application. Sweet cookies contain fat, sugars, and wheat flour [3]. Cookies or biscuits are products obtained by mixing flour, starch, and/or other ingredients, followed by kneading and baking. They can have various coatings, fillings, shapes, and textures. Wheat flour is the most important technological and commercial component in cookie making because it imparts the desired texture characteristics to the baked product. It forms the basis of the formulation, and additional ingredients may be included in the cookie dough, providing characteristics of softness, texture, and flavor [4,5].

The growth of the cookie market is a challenge in the bakery industry, which should meet the consumers’ demand with competitive products, food safety, and a longer shelf life, as well as enabling sustainability projects and reducing production losses [6]. Using waste or products not up to visual standards may be an economic strategy for companies looking for solutions to reduce costs and environmental impacts. In this context, investments in technologies for using by-products in production lines are necessary, and aim to add value to existing products on the market without losing the sensory characteristics of the final product [4].

In cake processing, the waste is generated at various stages of the baking process, including unmolding and fragmenting, filling injection, icing application, and primary packaging. These non-standard cakes are normally considered as waste and cannot be reused as the same product without new processing. For this reason, this study brings a creative approach to reducing food waste through upcycling cake waste from automated baking production into cookies. Therefore, this study aimed to evaluate the potential of three types of industrial cake waste (baked basic mini cake (BMC), filled mini cake (FMC), and traditional mini cake (TMC)) from a bakery automatic production line as sustainable alternatives for wheat flour replacement in ring-shaped cookies. After initial screening, FMC in different concentrations was used for cookie production, and its physical characteristics and physicochemical properties were evaluated over 150 days of storage.

## 2. Materials and Methods

**Figure 1:**
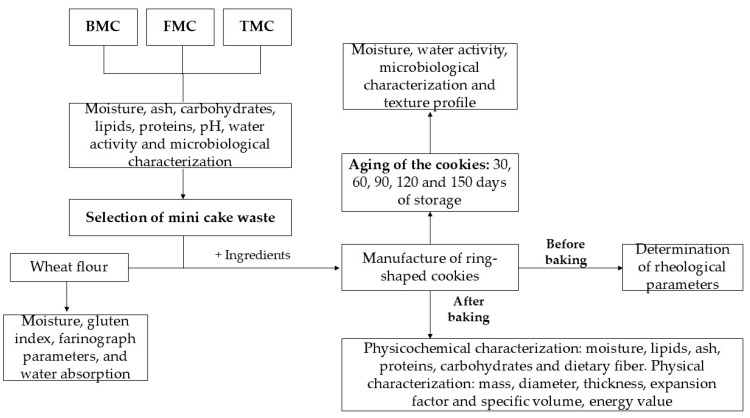
Flowchart of the processing steps. Legend: BMC = basic mini cake; FMC = filled mini cake; TMC = traditional mini cake.

The flowchart of the various processing steps, including baked mini cake waste, raw materials, processing conditions, and ring-shaped cookie determinations are shown in Figure 1.

### 2.1. Collection and Characterization of Mini Cake Waste

Baked mini cake waste was collected from an automated baking production line in the state of Parana, Brazil (latitude −25.3658014 and longitude −51.4865002). Uneven cakes in shape and size were used in the study, as follows: basic (BMC), filled (FMC), and traditional (TMC) mini cakes. Industrially, cakes had similar ingredients with different additives. BMC samples had a small amount of filling (consisting of sugar and fat) compared to their total mass after baking. The waste was collected in duplicate on different production days and stored at 15 °C for up to 2 days. The moisture, ash, carbohydrates, lipids, crude protein contents, aw, and pH values of mini cake waste were analyzed in triplicate [7]. The microbiological characterization consisted of the presence of *Salmonella* sp. per 25 g and the enumeration of coagulase-positive staphylococci per gram [8].

### 2.2. Wheat Flour Analysis

The moisture content was determined after oven-drying to constant weight at 105 °C. The gluten content was determined using a Glutomatic apparatus (Peter Instruments^®^, Hagersten, Sweden), and the wet gluten content was expressed as a percentage of the sample on a 14% moisture basis, calculating the ratio between total wet gluten per gram and 100% moisture in the flour [9]. The water absorption index was determined according to [10]. The farinograph parameters were determined in a farinograph (Brabender^®^ No. 174509, model 820600, Duisburg, Germany) according to method 54-21.02. The farinograph parameters were the arrival time, water absorption, dough stability, dough development time, and mixing tolerance index [9].

### 2.3. Manufacture of Ring-Shaped Cookies

Cookies were formulated with a fixed percentage of crystal sucrose (19.71%), drinking water (14.44%), hydrogenated vegetable fat (7.88%), cassava starch (3.29%), cocoa powder (2.04%), invert sugar (1.64%), soy lecithin (0.65%), sodium chloride (0.33%), ammonium bicarbonate (0.30%), sodium bicarbonate (0.26%), and chocolate flavoring (0.20%). Six cookie formulations were made with variations in the percentage of wheat flour and FMC waste. Before use, the FMC was ground in an industrial mixer to standardize the particle size and avoid the formation of lumps. Wheat flour was replaced with 10, 15, 20, 30, and 50% (*w*/*w*) of FMC, and the formulations were named T1 (standard—0%), T2 (10%), T3 (15%), T4 (20%), T5 (30%), and T6 (50%).

The ring-shaped cookies were made according to the standard AACC method 10-50D (Ref. [9]. For each formulation, the dough was prepared in a Kitchenaid^®^ Professional 5 Plus mixer, United States, for 8 min in phase 1 (mixing liquid and pasty ingredients) and for 2 min in phase 2 (adding dry ingredients). The cookies (6.5–7.5 g of dough each) were then manually shaped using a cookie ring approximately 35 mm in diameter and 7 mm high. After shaping, the cookies were baked in a Millenium 1CC ELT Perfecta^®^ São Paulo, Brazil, oven at 180 °C for 12 min. After baking, they were cooled to 25 °C for 20 min and wrapped in high-barrier metallized bioriented polypropylene (BOPP) film and stored at 25 ± 1 °C in a light-protected environment.

### 2.4. Rapid Viscosity Analysis (RVA) of Cookie Dough Before Baking

The cookie dough (before molding and baking) was evaluated in triplicate by RVA STD1 (Rapid Visco Analyzer, RVA-4500^®^, Hagersten, Sweden), using the Thermocline software TCW3 v 3.0 for Windows^®^. The analysis was carried out with 3.5 g of cookie dough at 14% moisture and dissolved in 25 mL of deionized water. Dispersion was carried out using the device’s acrylic stirrer. The pasting temperature, peak viscosity, setback viscosity, breakdown viscosity, final viscosity, and setback were evaluated.

### 2.5. Characterization of Ring-Shaped Cookies After Baking

Moisture content, lipids, ash, crude protein, carbohydrates, and pH were assayed [7] for the baked ring-shaped cookies. Physical parameters, namely mass, diameter, and thickness (Mitutoyo^®^ Vernier caliper, Kanagawa, Japan) [9], expansion factor (ratio of the diameter to the thickness), and specific volume (ratio of the apparent volume to the mass after cooking) (cm^3^g^−1^) were also determined after cookies baking. The physicochemical analyses were carried out at least in triplicate, and the physical analyses were carried out with 20 repetitions.

The energy value and the nutritional tables of the different formulations were calculated according to Atwater’s conversion values, multiplying the carbohydrate (except fiber) and protein content by 4 and lipids by 9 [11]. The nutrition labeling was based on the physicochemical characterization according to the rules established by current Brazilian legislation for packaged foods [12]. For comparison purposes, the recommended portion was based on the portion of commercial analog products (30 g), a reference value used for nutritional table calculation.

### 2.6. Ageing of Ring-Shaped Cookies

The ring-shaped cookies were packaged in high-barrier metallized bioriented polypropylene (BOPP) film, and the aging of the cookies was assessed over a period of 5 months, at intervals of 30, 60, 90, 120, and 150 days of storage at 25 °C, through the analysis of texture, moisture content [7], water activity (a_w_) (25 °C, Novasina Labmaster^®^ apparatus, Lachen, Sweden), and the presence of *Salmonella* sp. in 25 g of sample and positive coagulase staphylococci per gram of sample [8]. The texture analysis was carried out using a TAXT2i texture analyzer (Stable Micro Systems^®^, Surrey, United Kingdon) with a 3-point bend rig (HDP/3PB) probe and HDP/90 platform [13]. The moisture content and a_w_ were performed with 5 repetitions, the texture profile was evaluated in at least 20 repetitions, and the microbiological characterization was carried out only once.

### 2.7. Data Analysis

All results were analyzed using analysis of variance (ANOVA), and the means were compared by Tukey’s test at a 5% probability level using the Sisvar ^®^ version 5.7 and Microsoft Excel^®^ software 2021 v 16.0. Principal component analysis (PCA) was conducted to understand the role of variables on the ring-shaped cookies using the statistic program Past^®^ version 4.03.

## 3. Results and Discussion

### 3.1. Characterization of Cake Waste and Wheat Flour

BMC waste had the highest moisture content (18.77%) (*p* < 0.05), and the lowest moisture contents were observed for FMC and TMC (14.18 and 14.87%, respectively) (Table 1). These differences in moisture content are due to the individual formulations of each mini-cake, since the dough-making, shaping, and baking steps have already been standardized on the manufacturing line. Also, due to the operational processing conditions, all mini cake waste types had similar physical and chemical characteristics to the regular cakes that were not physically defective during manufacture. The BMC, FMC, and TMC samples exhibited lower moisture contents when compared to the cakes made with preservatives, such as calcium propionate and sorbic acid (20.92 and 21.57%, respectively). These preservatives allow for the production of moist but safe products [14].

The lipid and protein contents were lower (*p* < 0.05) in TMC (17.39 and 4.98%, respectively) when compared to BMC and FMC, also probably due to its formulation. The ash contents ranged from 1.04% (BMC) to 1.25% (FMC), which is lower than that observed by other authors for the blends of garri flour, soybean cake flour, and millet flour used to make a potentially functional dough (3.1%) [15]. The ash content is related to the composition of the ingredients in the formulations, as products containing whole wheat flour or brown sugar may have a higher mineral content than cakes made with traditional ingredients and white wheat flour.

The carbohydrate contents ranged from 50.87 to 61.67% (BMC and TMC, respectively), with FMC having the highest content among mini cakes (*p* < 0.05). These results were lower than those observed for cakes made with whole wheat flour and soya cake, which ranged from 73.73 to 74.06% [15].

No differences (*p* < 0.05) were observed for the pH values of the samples, with values of 6.86, 6.84, and 6.89 for BMC, TMC, and FMC, respectively. The mini cakes pH values were similar to those found in the literature [14,16]. The a_w_ values showed some variation (*p* < 0.05) in the BMC and TMC samples (0.76 and0.75) and FMC (0.72); this lower a_w_ could be related to its lower moisture content (14.18%). Physicochemical parameters, such as a_w_ and pH, are directly related to the microbiological deterioration of bakery products, particularly breads and cakes. Products with an intermediate a_w_ (0.6–0.85) are generally more prone to such deterioration [14]. Consequently, upcycled mini cake waste intended for use in new formulations should be refrigerated and properly packaged to ensure its quality as a raw material. Microbiological analyses showed the absence of *Salmonella* sp. in 25 g and 1.0 × 10^1^ CFU/g for positive coagulase staphylococci, which is in accordance with the current Brazilian regulation [17].

Based on the physicochemical characterization of the mini cake wastes (Table 1) and microbial safety, they all present valuable potential for upcycling in new product development, helping to reduce food waste. The low moisture content and a_w_ of TMC and FMC make them suitable as shelf-stable raw materials. Only FMC was used to produce ring-shaped cookies, as its moisture content (14.18%) closely matched wheat flour (13.85%), eliminating the need for an additional mass balance step. Given the chemical composition of each mini cake waste type, we believe that the results from ring-shaped cookies can be extrapolated to using other cake wastes as raw materials.

It is important to highlight the wheat flour quality for cookies’ development. The wheat flour used in the experiments had a moisture content of 13.85%, within the limit set by Brazilian legislation (15%) [18]. Dry gluten, wet gluten, and gluten index contents were 10, 28.6, and 97.4%, respectively, which were considered strong and recommended for cookie production. Gluten indicates that the flour contains high levels of insoluble proteins, and it provides structure for baking products [19].

Water absorption is the amount of water flour takes to achieve the desired consistency. The flour used in the experiments had a water absorption of 58.9%, and the dough development time was 14.67 min, the period needed after mixing flour dough to reach its maximum consistency. This is a relevant factor when making bakery products using wheat flour. The mixing tolerance index was 30 min. In addition, in the manufacture of the cookies, the flour was partially replaced by cake waste, which can interfere with the product’s manufacturing characteristics, such as kneading and shaping.

### 3.2. Characterization of Ring-Shaped Cookie Dough Using Mini Cake Waste

During the cookie-making process, the formulations T5 and T6, made with 30 and 50% FMC waste in place of wheat flour, respectively, exhibited a non-adequate texture for ring-shaped molding. The cookies did not retain their shape during molding because the dough lacked sufficient firmness. Therefore, they were discarded. While the cookies were molded manually, trained researchers carried out the process and checked the moldability parameter qualitatively. Promising results were reported by adding wheat bran at 20, 30, and 40% as a substitute for wheat flour in cookie production, resulting in good-quality baked cookies. However, adding 40% wheat bran negatively affected the cookies’ quality, providing a very hard texture [20]. Based on the qualitative molding behavior of the dough of this study, only the standard (T1) and the formulations T2, T3, and T4 were selected, which contained 0, 10, 15, and 20% of FMC waste as a substitute for wheat flour, respectively.

No changes (*p* > 0.05) were observed in the paste temperature between the cookies’ doughs (Table 2). The T2, T3, and T4 formulations had similar viscosity characteristics, which allows for process standardization by choosing the percentage of waste to be used as a partial substitute for wheat flour.

The highest viscosity peak (*p* < 0.05) during heating was observed for the standard formulation T1 (248 cP), made without the addition of cake waste (Table 2). This result may indicate the presence of starch granules with more uniform swelling properties during heating in RVA. When starch is heated with excess water, the granules absorb water, and the crystalline organization is disrupted, forming amorphous structures. This molecular disorganization results in starch gelatinization, characterized by a rapid increase in viscosity, typically occurring within 3 to 5 min under appropriate heating conditions [21].

The standard (T1) formulation had the highest (*p* < 0.05) peak viscosity (248.00 cP). A decrease in this parameter was observed with the gradual addition of FMC waste, and the lowest peak viscosity (124.67 cP) was observed for formulation T4, containing 20% FMC waste as a substitute for wheat flour. On the other hand, the peak time and paste temperature (Table 2) did not differ between the formulations (*p* < 0.05). The paste viscosity, time, and temperature are relevant when sizing industrial equipment. The addition of cake waste to the cookie dough led to a decrease in the paste viscosity. Its use can be adapted for the same equipment used to make traditional wheat flour-based cookies.

The standard (T1) formulation exhibited the highest breakdown viscosity (80.67 cP) (*p* > 0.05) (Table 2), indicating lower stability during heating. In contrast, the breakdown viscosity of formulations T2, T3, and T4 were not different from each other (*p* > 0.05), suggesting that adding FMC waste to the dough enhanced paste viscosity stability compared to the standard (T1) formulation. This behavior is due to breakdown viscosity, which indicates the collapse of the starch granule structure and is caused by the rupture of swollen granules. This parameter is associated with the starch’s heat stability and ease of cooking [22].

The final viscosity of the standard (T1) formulation was higher than that of the other samples (*p* < 0.05), with T3 and T4 showing the lowest final viscosity (Table 2). The decrease in final viscosity can be attributed to the reorganization of amylose, lipids, and protein molecules, forming a weaker starch paste during cooling [23]. Another hypothesis is that the FMC waste experienced some starch molecular degradation during its previous baking, mainly due to a decrease in the average length of amylose, which reduced the final viscosity [24].

These results suggest that the greater the addition of FMC waste, the lower the tendency for starch retrogradation. When assessing the overall behavior of starch retrogradation, it can be hypothesized that incorporating FMC waste into cookie dough decreases starch reassociation during cooling, reduces dough viscosity, and produces a soft texture for bakery products. The starch retrogradation is probably also reduced or slowed down during the cookie’s storage.

### 3.3. Physical and Physicochemical Characterization of Ring-Shaped Cookies Before and After Baking

The dough weight was standardized across all formulations, averaging 6.62 g. The initial diameter (before baking) of the standard (T1) formulation was smaller than that of the other formulations (*p* < 0.05) (Table 3). This difference may be related to the texture of the dough and its ease of molding, as the cookies containing FMC waste exhibited a stickier dough and may have deformed during arrangement on the baking sheets. A similar trend was observed in the final diameter of the baked ring-shaped cookies. After baking and cooling, the final weight of the cookies varied from 5.14 g (T3) to 5.40 g (T4) (Table 3). Although there was a difference between the samples, in practice, this variation is very small and is inherent to the manufacturing process.

The formulations T2, T3, and T4 showed a higher volume expansion index when compared to the standard T1 (Table 3). However, this result did not affect the specific volumes of the cookies, which did not differ from each other (*p* > 0.05) and averaged 2.29 cm^3^g⁻^1^. These findings indicate that the ring-shaped cookies exhibited uniform size after baking despite the slight variation in initial diameter. It is worth mentioning that the experiments were conducted on a laboratory scale, with established industrial processing, trained analysts, and calibrated instruments. This is relevant to ensure the reproducibility of the results and to allow easier scale-up.

Studies on rice and wheat bran cookies reported lower volume expansion indices (2.04 to 3.09) and specific volumes (1.2 to 1.63 cm^3^g^−1^), respectively. Both the ingredients in the formulations and the baking conditions directly influence the physical properties of the raw dough and the baked cookies [25].

The physicochemical characterization of the baked cookies is described in Table 3. The standard (T1) formulation had the highest moisture content (7.26%) when compared to the other formulations (*p* < 0.05), likely due to the interaction between water and the ingredients. Adding FMC waste as a replacement for wheat flour increased the dietary fiber content, which may enhance water absorption due to the hydrophilic nature of the fibers. The lipids from FMC waste can reduce water retention in the dough and may lead to lower moisture content in the baked product [26]. Another compound that may be related to lower moisture content is the shorter amylose length in FMC waste formed due to the starch degradation from cake baking [27].

A higher lipid content (*p* < 0.05) was observed for the T4 formulation (13.59%), while the standard formulation T1 exhibited the lowest lipid content (11.27%) (Table 3). This result was expected, as the FMC waste originally contained fats in its formulation. The protein and carbohydrate contents did not differ between the formulations (*p* < 0.05), with average values of 7.44 and 69.27%, respectively. The ash contents of the cookies ranged from 1.29 (T1) to 1.42% (T4), with no difference between the formulations (*p* > 0.05). Concerning the dietary fiber, the standard formulation T1 had a lower content (2.72%) when compared to the other formulations (*p* < 0.05). Thus, the use of FMC waste in manufacturing the ring-shaped cookies contributed to an increase in the fiber content of the product, which can be attractive considering the technological processing.

The nutritional information on food products represents an important aspect for consumers. Thus, a simulation of the nutritional value table was performed on the cookies developed in our study (Table 4). The energy value was similar for all formulations, ranging from 123 to 128 Kcal for the standard (T1) and 20% (T4) samples, respectively. The T4 formulation had a total fat content of 4.1 g, which is 22% higher than the standard (T1) formulation (3.4 g). For the other parameters, the difference between the nutritional components of the standard (T1), 10% (T2), and 15% (T3) formulations was less than 20%, which is the tolerance allowed by Brazilian legislation for the nutrient values declared on the labels. Thus, the standard T1 and the T2 and T3 formulations could be packaged in a standard package, respecting the other legal aspects of food labeling.

### 3.4. Aging Analysis of Ring-Shaped Cookies

The moisture contents of all samples varied during the 150 days of storage (*p* < 0.05) (Table 5). The standard (T1) formulation consistently showed the highest moisture content, followed by formulations T2, T3, and T4. The dietary fibers from FMC waste (T3 and T4) may be responsible for the lower moisture content during storage (Table 5). The binding water slows the staling process by reducing water migration from the crumb to the crust during cookie storage [27].

Similarly, although there was a slight variation in a_w_ throughout storage, it remained below 0.48 across all samples, with T1 exhibiting the highest a_w_. Moisture content and water activity (a_w_) are critical parameters when studying the aging of cookies, as they are related to product preservation during transportation and packaging. To ensure the extended shelf life, the a_w_ of cookies must be below 0.6 to guarantee the microbiological and sensory stability of the product [28]. Microbiological analyses confirmed the absence of *Salmonella* sp. in 25 g and positive coagulase staphylococci counts of 1.0 × 10^1^/g throughout the 150-day storage period.

Based on the moisture content, a_w_, and microbiological parameters, the ring-shaped cookies can be stored for at least 150 days at 25 ± 1 °C in bioriented polypropylene (BOPP) packaging. In addition, factors, such as good manufacturing practices, proper baking and cooling times, and appropriate packaging, play essential roles in maintaining cookie quality.

**Figure 2:**
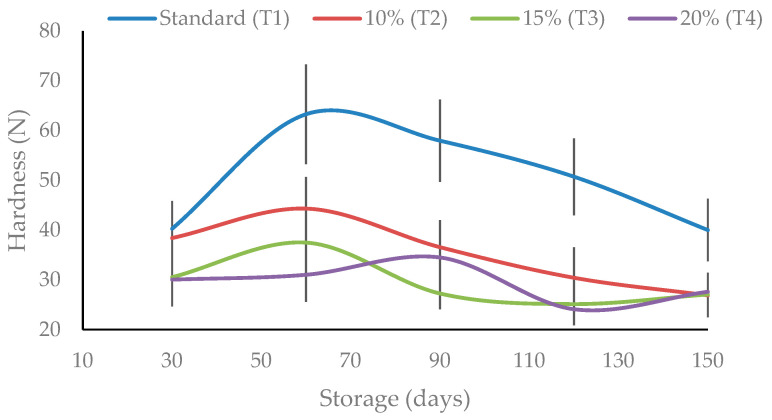
Hardness of ring-shaped cookies for standard (T1), 10% (T2), 15% (T3), and 20% (T4) FMC waste samples during 150 days of storage.

The cookie formulation affected the texture profile during storage (Figure 2). A general tendency toward a softer texture was observed for the formulations augmented with FMC waste compared to the standard T1 sample. After 30 days of storage, the hardness of all treatments was similar, ranging from 30 N to 40 N (Figure 2). However, by 60 days, T1 showed increased hardness, while formulations T2, T3, and T4 maintained lower hardness, a trend that persisted up to 150 days (Figure 2). This suggested that FMC waste used as a partial substitute for wheat flour worked as an emulsifier, resulting in cookies with a soft texture. This may be due to lipids, carbohydrates, and some emulsifiers, moisturizers, and thickeners in the FMC waste. This is also consistent with the rheological behavior of the raw cookie dough observed through RVA analysis (Table 2).

In addition to the presence of lipids and carbohydrates, the texture of the cookies can be influenced by the quality of the flour, the moisture content of the product, and the storage conditions [29]. These parameters were standardized in our study, supporting our hypothesis of the FMC waste emulsifying effect. Notably, even though the moisture content was lower in formulations with FMC waste compared to standard (T1) samples (Table 5), these cookies exhibited reduced hardness after 150 days of storage (Figure 2). This may be due to a reduced staling effect, as lipids formed a barrier that prevented moisture migration from the crumb to the crust. Additionally, lipids, dextrin, and sugars in FMC waste likely reduced starch retrogradation, further contributing to a softer texture [27].

Replacing wheat flour with millet flour at different concentrations (0, 20, 40, 60, 80, and 100%) in the cookie processing reduced cookie hardness with the increase in the millet flour concentration. This probably happens due to the dilution of the gluten content [30]. Incorporating oats (25, 35, and 45%) into the texture parameters of the cookie dough decreased the dough hardness from 25.05 to 21.73 N for dough made with 0 to 25% oats, with no additional changes for the other concentrations [31]. Generally, the texture results found in this study are consistent with the texture profile reported in the literature.

**Figure 3:**
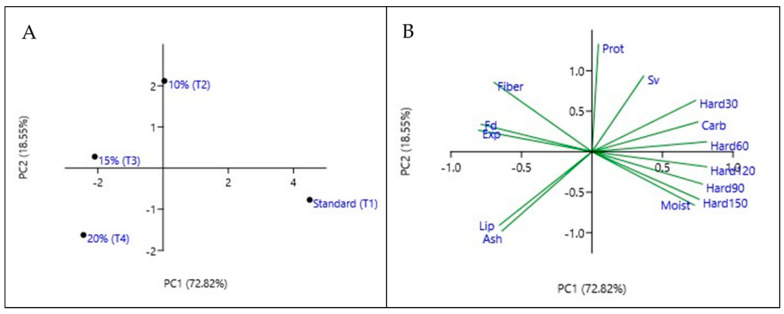
Principal component analysis (PCA) of physical and physicochemical ring-shaped cookies characteristics. Left (**A**) is the quadrant and right (**B**) is the variables’ grouping.

Principal component analysis (PCA) was performed in order to better understand the relationship between the experiments carried out with different formulations of ring-shaped cookies (Figure 3A,B). The response variables evaluated were the measures of final diameter (Fd), expansion factor (Exp), specific volume (Sv), moisture (Moist), ash, lipids (Lip), protein (Prot), dietary fiber (Fiber), and carbohydrate (Carb) contents, and hardness after 30, 60, 90, and 150 days of storage (Hard). These variables were represented by PC1 and PC2, corresponding to 91.37% of the total data variability. The data plotted in Figure 3A showed that the standard (T1) treatment was positioned in the right quadrant and was the sample responsible for the highest moisture content, hardness at time, and carbohydrate content (Figure 3B). At the same time, treatments T2 (10%), T3 (15%), and T4 (20%) demonstrated similarity and are positioned in the left quadrant. The separation of the variables along PC1 was influenced by the analyses of lipids, ash, moisture, hardness, carbohydrates, dietary fiber, expansion factor, and final diameter, while PC2 was influenced mostly by protein and specific volume. The 10% (T2) and 15% (T3) samples are correlated for the analyses of protein, dietary fiber, and expansion factor and final diameter, while the 20% T4 sample is correlated with the ash and lipid responses only (Figure 3B).

## 4. Conclusions

Cake waste from different products (BMC, FMC, and TMC) has a similar physicochemical composition and can be upcycled as a new product. Ring-shaped cookies made with 10, 15, and 20% cake waste (FMC) as a partial substitute for wheat flour have proven to be a promising approach, according to their physical aspects and the physicochemical and microbiological characterization. The moisture content, a_w_, microbiological parameters, and texture profile of the formulations showed that the ring-shaped cookies could be stored in bioriented polypropylene (BOPP) packaging for 150 days at 25 ± 1 °C in the dark. From the point of view of process standardization, the formulations made with 10 and 15% cake waste can be packaged in the same type of packaging without altering the nutritional tables. The use of cake waste that was originally discarded resulted in cookies with technological and stable qualities for at least 150 days of storage and contributes to the sustainable production of food, offering economic and environmental benefits.

## Figures and Tables

**Table 1:** Characterization of mini cake waste from the bakery production line.

Waste ^1^	Moisture (%)	Ash (%)	Lipids (%)	Protein (%)	Carbohydrates (%)	pH	a_w_
BMC	18.77 ± 0.49 ^a^	1.04 ± 0.10 ^b^	23.36 ± 2.83 ^a^	5.95 ± 0.09 ^a^	50.87	6.86 ± 0.19 ^a^	0.76 ± 0.01 ^a^
TMC	14.87 ± 1.41 ^b^	1.20 ± 0.08 ^ab^	17.39 ± 0.35 ^b^	4.98 ± 0.17 ^b^	61.67	6.84 ± 0.12 ^a^	0.75 ± 0.00 ^a^
FMC	14.18 ± 0.33 ^b^	1.25 ± 0.04 ^a^	20.88 ± 0.68 ^ab^	6.05 ± 0.05 ^a^	58.33	6.89 ± 0.24 ^a^	0.72 ± 0.01 ^b^

^1^ Means of three repetitions±standarddeviation Averages followed by the same letters in the corresponding columns do not differ statistically from each other using the Tukey test at the 5% significance level. BMC = basic mini cake; FMC = filled mini cake; TMC = traditional mini cake.

**Table 2:** Rapid viscosity analysis (RVA) of standard dough and dough made with FMC waste.

Formulation ^1^	Peak (cP)	Minimum (cP)	Breakdown (cP)	Final (cP)	Tendency to Retrogradation (cP)	Peak Time (min)	Paste Temperature (°C)
Standard (T1)	248.00 ± 26.06 ^a^	167.33 ± 10.21 ^a^	80.67 ± 16.26 ^a^	519.33 ± 35.35 ^a^	352.00 ± 25.51 ^a^	5.56 ± 0.10 ^a^	94.92 ± 0.20 ^a^
10% (T2)	169.33 ± 3.51 ^b^	124.67 ± 2.08 ^b^	44.67 ± 1.53 ^b^	395.67 ± 4.62 ^b^	271.00 ± 2.65 ^b^	5.44 ± 0.08 ^a^	94.73 ± 0.11 ^a^
15% (T3)	141.33 ± 5.03 ^bc^	108.00 ± 3.61 ^c^	33.33 ± 2.08 ^b^	346.33 ± 2.31 ^bc^	238.33 ± 1.53 ^bc^	5.38 ± 0.10 ^a^	94.73 ± 0.03 ^a^
20% (T4)	124.67 ± 3.51 ^c^	98.33 ± 2.89 ^c^	26.33 ± 1.53 ^b^	302.67 ± 7.37 ^c^	204.33 ± 5.86 ^c^	5.36 ± 0.10 ^a^	94.88 ± 0.19 ^a^

^1^ Means of three repetitions ± standard deviation. Averages followed by the same letters in the corresponding columns do not differ statistically from each other using the Tukey test at the 5% significance level. FMC = filled mini cake.

**Table 3:** Physical aspects and physicochemical characterization of ring-shaped cookies from Formulations T1, T2, T3 and T4 before and after baking.

Formulation ^1^	Physical Characteristics Before Baking		Physical Characteristics After Baking			
	Initial Weight (g)	Initial Diameter (cm)	Final Weight (g)	Final Diameter (cm)	Expansion Factor	Specific Volume (cm^3^ g^−1^)
Standard (T1)	6.61 ± 0.12 ^a^	3.43 ± 0.11 ^b^	5.17 ± 0.11 ^b^	3.73 ± 0.11 ^b^	3.45 ± 0.35 ^c^	2.31 ± 0.27 ^a^
10% (T2)	6.67 ± 0.22 ^a^	3.60 ± 0.09 ^a^	5.20 ± 0.19 ^b^	4.07 ± 0.12 ^a^	4.28 ± 0.31 ^b^	2.38 ± 0.19 ^a^
15% (T3)	6.60 ± 0.16 ^a^	3.55 ± 0.08 ^a^	5.14 ± 0.13 ^b^	4.07 ± 0.18 ^a^	4.66 ± 0.34 ^a^	2.22 ± 0.21 ^a^
20% (T4)	6.61 ± 0.19 ^a^	3.54 ± 0.8 ^a^	5.40 ± 0.16 ^a^	4.12 ± 0.12 ^a^	4.48 ± 0.36 ^ab^	2.27 ± 0.14 ^a^
**Formulation ^1^**	**Physicochemical Characteristics**					
	**Moisture (%)**	**Ash (%)**	**Lipids (%)**	**Protein (%)**	**Dietary Fiber (%)**	**Carbohydrates (%)**
Standard (T1)	7.26 ± 0.22 ^a^	1.29 ± 0.01 ^a^	11.27 ± 0.73 ^b^	7.36 ± 0.25 ^a^	2.72 ± 0.01 ^d^	70.10 ± 0.80 ^a^
10% (T2)	3.97 ± 0.16 ^c^	1.30 ± 0.04 ^a^	11.60 ± 0.77 ^b^	7.71 ± 0.66 ^a^	5.74 ± 0.01 ^a^	69.68 ± 1.41 ^a^
15% (T3)	4.49 ± 0.17 ^b^	1.35 ± 0.03 ^a^	12.33 ± 0.59 ^ab^	7.80 ± 0.88 ^a^	5.49 ± 0.01 ^b^	68.54 ± 1.60 ^a^
20% (T4)	4.33 ± 0.13 ^bc^	1.42 ± 0.08 ^a^	13.59 ± 0.07 ^a^	6.90 ± 0.68 ^a^	4.97 ± 0.01 ^c^	68.79 ± 0.43 ^a^

^1^ Means of three repetitions ± standard deviation. Averages followed by the same letters in the corresponding columns do not differ statistically from each other using the Tukey test at the 5% significance level. FMC = filled mini cake.

**Table 4:** Nutritional information on ring-shaped cookie formulations.

Nutrition Facts	Standard (T1)		10% (T2)		15% (T3)		20% (T4)	
	* Per Serving (30 g)	%DV	* Per Serving (30 g)	%DV	* Per Serving (30 g)	%DV	* Per Serving (30 g)	%DV
Energy value	123 kcal = 520 kJ	6	125 kcal = 526 kJ	6	126 kcal = 526 kJ	6	128 kcal = 538 kJ	6
Carbohydrates (g)	21	7	21	7	20.6	7	20.6	7
Proteins (g)	2.2	3	2.3	3	2.3	3	2.1	3
Total fat (g)	3.4	6	3.5	6	3.7	7	4.1	7
Saturated fat (g)	0.7	3	0.8	4	0.8	4	0.9	4
Trans fat (g)	0.9	0.4	0.9	0.4	0.9	0.4	0.9	0.4
Dietary fiber (g)	0.8	3	1.7	6	1.6	6	1.5	5
Sodium (mg)	65	3	67	3	68	3	69	3

* One portion = 30 g. %DV based on a 2000 kcal diet.

**Table 5:** Moisture content and water activity (a_w_) of ring-shaped cookies T1, T2, T3 and T4 during 150 days of storage.

Storage ^1^ (Days)	Standard (T1)		10% (T2)		15% (T3)		20% (T4)	
	Moisture (%)	A_w_	Moisture (%)	A_w_	Moisture (%)	A_w_	Moisture (%)	A_w_
0	7.26 ± 0.22 ^a^	-	3.97 ± 0.16 ^c^	-	4.49 ± 0.17 ^bc^	-	4.33 ± 0.13 ^d^	-
30	5.09 ± 0.02 ^d^	0.35 ± 0.01 ^b^	6.36 ± 0.04 ^a^	0.42 ± 0.01 ^a^	4.30 ± 0.06 ^c^	0.31 ± 0.02 ^a^	5.27 ± 0.03 ^c^	0.36 ± 0.01 ^a^
60	6.43 ± 0.48 ^c^	0.37 ± 0.06 ^b^	6.77 ± 0.23 ^a^	0.35 ± 0.01 ^b^	6.48 ± 0.30 ^a^	0.33 ± 0.00 ^a^	7.05 ± 0.41 ^a^	0.33 ± 0.00 ^b^
90	8.14 ± 0.26 ^a^	0.48 ± 0.00 ^a^	6.49 ± 0.04 ^a^	0.39 ± 0.00 ^a^	5.80 ± 0.39 ^ab^	0.34 ± 0.01 ^a^	5.93 ± 0.14 ^b^	0.34 ± 0.01 ^b^
120	7.40 ± 0.04 ^b^	0.48 ± 0.01 ^a^	5.54 ± 0.07 ^b^	0.39 ± 0.01 ^a^	4.45 ± 0.51 ^bc^	0.33 ± 0.01 ^a^	5.21 ± 0.06 ^c^	0.36 ± 0.01 ^a^
150	7.79 ± 0.15 ^ab^	0.47 ± 0.00 ^a^	6.38 ± 0.78 ^a^	0.41 ± 0.04 ^a^	4.51 ± 1.24 ^bc^	0.33 ± 0.07 ^a^	4.82 ± 0.11 ^d^	0.32 ± 0.01 ^c^
* Mean value	7.02 ±1.11	0.43 ± 0.06	5.92 ±1.04	0.39 ± 0.03	5.00 ±0.91	0.33 ± 0.01	5.43 ±0.95	0.34 ± 0.02

^1^ Values expressed as the mean of five replicates ± standard deviation. Averages followed by the same letters in the corresponding columns do not differ statistically using the Tukey test at the 5% significance level. - No measurements at time 0. * Mean and standard deviation considering storage times from 30 to 150 days.

## Data Availability

The original contributions presented in this study are included in the article. Further inquiries can be directed to the corresponding authors.
